# Fucoidan induces Toll-like receptor 4-regulated reactive oxygen species and promotes endoplasmic reticulum stress-mediated apoptosis in lung cancer

**DOI:** 10.1038/srep44990

**Published:** 2017-03-23

**Authors:** Hsien-Yeh Hsu, Tung-Yi Lin, Mei-Kuang Lu, Pei-Ju Leng, Shu-Ming Tsao, Yu-Chung Wu

**Affiliations:** 1 Department of Biotechnology and Laboratory Science in Medicine, National Yang-Ming University, Taipei, Taiwan; 2 Program in Molecular Medicine, National Yang-Ming University and Academia Sinica, Taipei, Taiwan; 3 The Genomics Research Center, Academia Sinica, Taipei, Taiwan; 4 National Research Institute of Chinese Medicine, Taipei, Taiwan; 5 Graduate Institute of Pharmacognosy, Taipei Medical University, Taipei, Taiwan; 6 Division of Thoracic Surgery, Department of Surgery, Taipei Veterans General Hospital, Taipei, Taiwan; 7 School of Medicine, National Yang-Ming University, Taipei, Taiwan

## Abstract

Fucoidan, a sulfated polysaccharide extracted from brown algae, exhibits anti-cancer activity. However, the effects and mechanism of fucoidan-induced apoptosis via endoplasmic reticulum (ER) stress is unclear. In this study, we demonstrated that fucoidan prevents tumorigenesis and reduces tumor size in LLC1-xenograft male C57BL/6 mice. Fucoidan induces an ER stress response by activating the PERK-ATF4-CHOP pathway, resulting in apoptotic cell death *in vitro* and *in vivo*. Furthermore, ATF4 knockdown abolishes fucoidan-induced CHOP expression and rescues cell viability. Specifically, fucoidan increases intracellular reactive oxygen species (ROS), which increase ATF4 and CHOP in lung cancer cells. Using the ROS scavenger *N*-acetyl-l-cysteine (NAC), we found that ROS generation is involved in fucoidan-induced ER stress-mediated apoptosis. Moreover, via Toll-like receptor 4 (TLR4) knockdown, we demonstrated that fucoidan-induced ROS and CHOP expression were attenuated. Our study is the first to identify a novel mechanism for the antitumor activity of fucoidan. We showed that fucoidan inhibits tumor viability by activating the TLR4/ROS/ER stress axis and the downstream PERK-ATF4-CHOP pathway, leading to apoptosis and suppression of lung cancer cell progression. Together, these results indicate that fucoidan is a potential preventive and therapeutic agent for lung cancer that acts via activation of ROS-dependent ER stress pathways.

Fucoidan consists of fucose-based sulfated polysaccharides and is extracted from brown algae; it is a heparin-like molecule with a substantial percentage of L-fucose and sulfated ester groups as well as small proportions of D-xylose, D-galactose, D-mannose, and glucuronic acid^1^,^2^,^3^. Fucoidan has low toxicity^4^ and various beneficial biological properties, such as anti-tumor, anti-bacterial and anti-inflammatory activities^5^. We recently reported that fucoidan leads to Smurf2-dependent ubiquitin proteasome pathway-mediated TGFbeta receptor (TGFR) degradation and inhibition of breast^6^ and lung cancer^7^ cell growth and mobility *in vitro* and *in vivo*. The molecular mechanisms of fucoidan-mediated antitumor signaling pathways have also been examined and proposed. In general, fucoidan appears to affect cancer cells through various biological processes or responses. One biological response may be accomplished by several independent signalling pathways. For example, a variety of signal transduction pathways can independently cause cellular apoptosis. The cellular and molecular biology aspects of fucoidan’s antitumor function include fucoidan-induced apoptosis, cell cycle arrest, anti-angiogenesis, inhibition of metastasis, migration and invasion, and immunological reactions^3^,^8^. In our current research, we examine fucoidan-induced apoptosis in lung cancer.

Lung cancer is one of the most common cancers in the world and has a high mortality rate. There are two types of lung cancer: non-small cell lung cancer (NSCLC, approximately 80% of lung cancers) and small cell lung cancer (SCLC, approximately 20% of lung cancers). Additionally, there are three types of NSCLC, including adenocarcinoma (~40% of NSCLCs), squamous cell carcinoma (~25–30% of NSCLCs), and large cell lung cancer (~10–15% of NSCLCs)^9^. Multiple anti-cancer drugs have been developed and used to inhibit tumor angiogenesis or induce cancer cell death via various signaling pathways^10^; however, these clinical drugs can result in tumor cell resistance and have numerous detrimental side effects^11^.

The endoplasmic reticulum (ER) is an important intracellular organelle with many functions, such as protein folding, initially post-translational modification, lipid biogenesis, and maintenance of Ca^2+^ homeostasis in cells^12^. Physiological or pathological conditions, such as hypoxia, nutrient deprivation and ER calcium depletion, interfere with the normal functions of the ER, resulting in accumulation of abnormal proteins. Induction of ER stress from accumulation of unfolded protein aggregates activate a series of intracellular signaling pathways known as the unfolded protein response (UPR)^13^. The UPR is activated by the coordinated action of three separate ER stress sensors that are located on the ER membrane including protein kinase RNA-like ER kinase (PERK); inositol-requiring protein-1α (IRE1α) and activating transcription factor 6 (ATF6)^14^. In general, PERK, a type III ER resident protein kinase, phosphorylates serine 51 of eukaryotic initiation factor 2α (eIF2α), and followed by the selective translation of ATF4, when cells are under ER stress^14^. ATF4 induces UPR target genes that promote ER folding and adaptation to stress via induction of C/EBP homologous protein (CHOP/GADD153)^15^. CHOP is a major pro-apoptotic transcription factor that increases the rate of *Bim* transcription^16^. It is reported that aberrant stimulation of ER or ER stress may imbalance the redox reactions and activate reactive oxygen species (ROS) release; moreover, ER stress–induced apoptosis is implicated in various pathological conditions including cancer^17^.

Reactive oxygen species (ROS) are defined as oxygen-containing, reactive chemical species. There are two types of ROS: free radicals and non-free radicals^18^. Free radical ROS, such as superoxide (O_2_^−^) and the hydroxyl radical (OH•), contain an unpaired electron. Non-free radical ROS do not have unpaired electrons but can be converted to free radical ROS^19^. ROS are produced by or derived from the mitochondria respiratory chain. In some cancer cells, ROS are produced through a reaction catalyzed by NADPH oxidase complexes^18^. Previous reports demonstrated that ROS may deplete calcium stores in the ER via inhibition of Ca^2+^-ATPase, leading to (or triggering) ER stress and apoptosis^20^. Additionally, ROS may also exacerbate protein misfolding in the ER lumen by oxidizing amino acids in folding proteins and inducing the UPR^21^, which then promotes ER stress.

Fucoidan-induced apoptosis of human MDA-MB-231 breast cancer cells and HCT116 colon cancer cells by modulating the ER stress cascades has been reported^22^. In addition, evidence of the involvement of ROS in a variety of fucoidan and induced apoptosis has been accumulated; for example, fucoidan of the Mozuku seaweed (*Cladosiphon novae*-*caledoniae* Kylin) activates a caspase-independent apoptotic pathway in human MCF-7 breast cancer cells through ROS-dependent JNK activation and the mitochondria-mediated Bcl-2 family pathways^23^; fucoidan (*Undaria pinnatifida* sporophylls) induces apoptosis in human SMMC-7721 hepatocellular carcinoma via the ROS-mediated mitochondrial pathway and activation of caspases process^24^; and fucoidan (*Fucus vesiculosus*) inhibits proliferation of human myelodysplastic syndrome/acute myeloid leukemia cell line SKM-1 via the activation of apoptotic pathways and production of ROS^25^. However, the mechanism by which fucoidan-induced extracellular signaling induces ER stress and ROS remains unclear. Therefore, we investigated the effect of fucoidan-mediated ROS (redox modulation) on induction of ER stress and the consequent apoptotic reactions in lung cancer.

In our current study, we demonstrate that fucoidan (i) promotes apoptosis of lung cancer cells, (ii) reduces lung tumorigenesis, (iii) induces ATF4 and CHOP protein expression in an LLC1-xenograft mice model via continuous oral feeding of fucoidan, and (iv) induces ROS-mediated ER stress via TLR4. Our study elucidates the mechanism underlying fucoidan-induced ROS-mediated induction of ATF4 and CHOP protein expression via the TLR4/ROS/PERK/eIF2α axes. Moreover, our findings suggest that fucoidan is a potential therapeutic agent for preventing lung tumorigenesis.

## Results

### Fucoidan prevents tumorigenesis in LLC1 cell-xenograft male C57BL/6 mice *in vivo*

**Figure 1:**
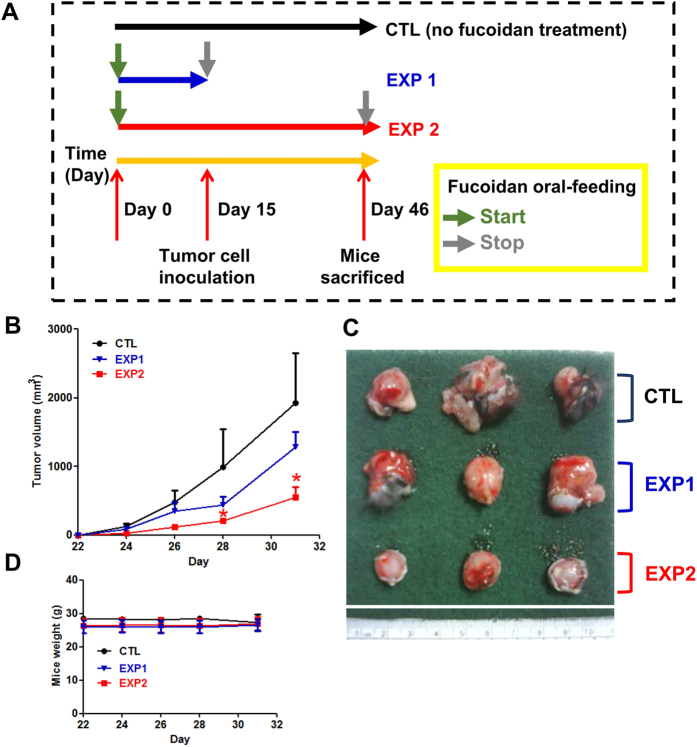
Fucoidan prevents tumorigenesis in LLC1-bearing C57BL/6 mice *in vivo*. Mice were pretreated with fucoidan (24 mg/kg) for 14 days before LLC1 cells (2 × 10^5^) were hypodermically inoculated into the dorsum of male C57BL6 mice. On the 15^th^ day, the mice were divided into three groups and processed in a “previous and continuous” fashion (**A**). In brief, the following groups were established: oral feeding with ddH_2_O (control), oral feeding with cessation of fucoidan feeding on the day of LLC1 cell inoculation, (EXP 1) and maintained fucoidan feeding for 26 days (EXP 2) at 7 oral feedings/week/mouse. (**B**) The tumor volumes and (**C**) tumor samples from the mice were collected at the end of the treatment. (**D**) The mouse weights were measured as indicated. Three of five individual experiments are presented (n = 5). Each bar represents the mean ± standard deviation (SD), and error bars indicate the SD. Statistically significant differences are shown (*P < 0.05) compared with the control group.

We previously demonstrated that continuous feeding with fucoidan effectively inhibits tumor growth in LLC1-bearing mice^7^. To further explore the cancer inhibitory activity of fucoidan, we examined the tumor volumes of mice that were exposed to oral feeding prior to hypodermic inoculation of cancer cells into the dorsum. Briefly, we divided the mice into three groups: group 1 was the ddH_2_O control (CTL); group 2 was fed only fucoidan prior to LLC1 inoculation, and oral feeding of fucoidan was stopped on the 15^th^ day (EXP 1); and group 3 was fed fucoidan for the entire experimental period (EXP 2; Fig. 1A). At the indicated time, the mice in EXP 1 exhibited smaller tumor volumes than those of the mice in CTL (Fig. 1B and C). Additionally, tumor volumes of mice in EXP 2 were significantly reduced compared with those of the mice in EXP 1 (Fig. 1B and C). Thus, fucoidan treatment in EXP 1 (i.e., discontinuous oral feeding with fucoidan) exhibited reduced efficacy in inhibiting tumor growth compared with that of the EXP 2 continuous fucoidan treatment. Moreover, a smaller difference was observed in the mouse body weights between the fucoidan-fed mice (EXP 1 and EXP 2) and the ddH_2_O controls (CTL; Fig. 1D). Together, these results indicate that continuous oral feeding with fucoidan elicits the greatest efficacy in preventing and suppressing tumorigenesis.

### Fucoidan reduces lung tumor growth and induces endoplasmic reticulum (ER) stress-related proteins in LLC1-bearing mice *in vivo* and *in vitro*

**Figure 2:**
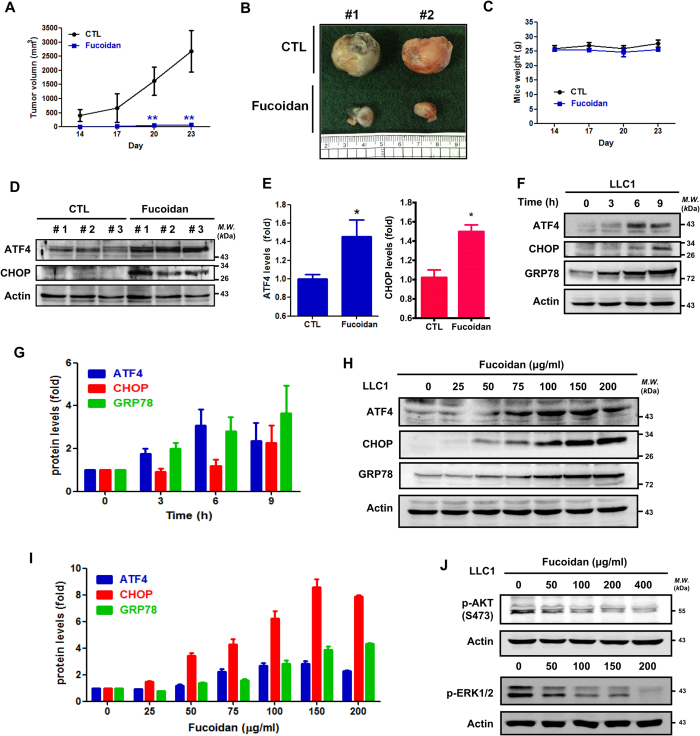
Fucoidan suppresses tumor growth and induces apoptosis-related proteins *in vitro* and *in vivo*. (**A**) Continuous once daily feeding of C57BL/6 male mice with fucoidan (24 mg/kg) for 14 days prior to inoculation with LLC1 cells. After prior oral administration of fucoidan, LLC1 cells were subcutaneously inoculated into the abdomens of mice, and continuous once daily fucoidan feeding was performed (24 mg/kg) for 23 days. The tumor volumes were measured every 3 days for 23 days. (**B**) Tumor samples from the mice were collected at the end of the treatment. (**C**) The body weights of the mice were measured every 3 days for 23 days. (**D**) After fucoidan treatment, the tumors were collected. The tumor samples were homogenized, and Western blotting analyses of tumor samples were performed to detect ATF4 and CHOP expression. (**E**) Quantification of fucoidan-mediated up-regulation of the apoptotic protein levels in (**D**). (**F**) LLC1 cells were treated with fucoidan (100 μg/ml) for 3–9 h followed by Western blotting analyses of whole-cell lysates to detect the expressions of the following ER stress-related proteins: ATF4, CHOP, and GRP78. (**G**) Quantification of fucoidan-mediated up-regulation of ER stress-related protein levels in (**F**). (**H**) LLC1 cells were treated with various dosages of fucoidan (μg/ml) for 24 h followed by Western blotting analyses of whole-cell lysates to detect ATF4, CHOP and GRP78 expression. (**I**) Quantification of fucoidan-mediated up-regulation of the ER stress-related proteins levels in (**H**). (**J**) LLC1 cells were treated with fucoidan (0 to 400 μg/ml) for 24 h followed by Western blotting analyses of whole cell lysates to detect p-Akt (Ser 473) and p-ERK1/2 expression. Actin was used as an internal control.

To investigate the effect of fucoidan feeding on LLC1-bearing mice *in vivo*, we fed the mice fucoidan (24 mg/kg/daily) for 14 days and subsequently hypodermically inoculated them with LLC1 cells in the dorsum on day 14. The treated mice were fed daily with fucoidan, and tumor growth rates were assessed over 23 days. The mice fed fucoidan exhibited significant reductions in tumor volumes (Fig. 2A,B). In contrast, a smaller difference was observed in body weights of the fucoidan-fed mice and the ddH_2_O control groups (Fig. 2C), which indicated this dose of fucoidan did not have a toxic effect in the C57BL/6 mice model. Next, the tumor lesions of the mice were collected to analyze ER stress-related protein expression. As shown in Fig. 2D and E, ATF4 and CHOP protein levels increased in the fucoidan-fed LLC1-bearing mice compared with those in the control group, indicating that fucoidan induced ER stress-related proteins in tumors *in vivo* (see the following section).

Further supporting the effect of fucoidan on LLC1 cells *in vitro*, we previously showed that fucoidan (effective concentration about 224 μg/ml) inhibits the proliferation of LLC1 cells in dose- and time-dependent manners using an MTT assay^7^. Specifically, we found that fucoidan induces ER stress-related protein expression, including GRP78, ATF4 and CHOP, in both time- and dose-dependent manners (Fig. 2F–I). Additionally, we demonstrated that fucoidan inhibits AKT and ERK phosphorylations (Fig. 2J), which are related to the viability and proliferation of cancer cells^26^. Together, these results indicate that induction of ER stress and inhibition of AKT/ERK signaling are involved in fucoidan-mediated prevention and suppression of tumorigenesis *in vitro* and *in vivo*.

### Fucoidan induces apoptosis-related reactions and p21 expression and inhibits AKT phosphorylation in human lung cancer *in vitro*

**Figure 3:**
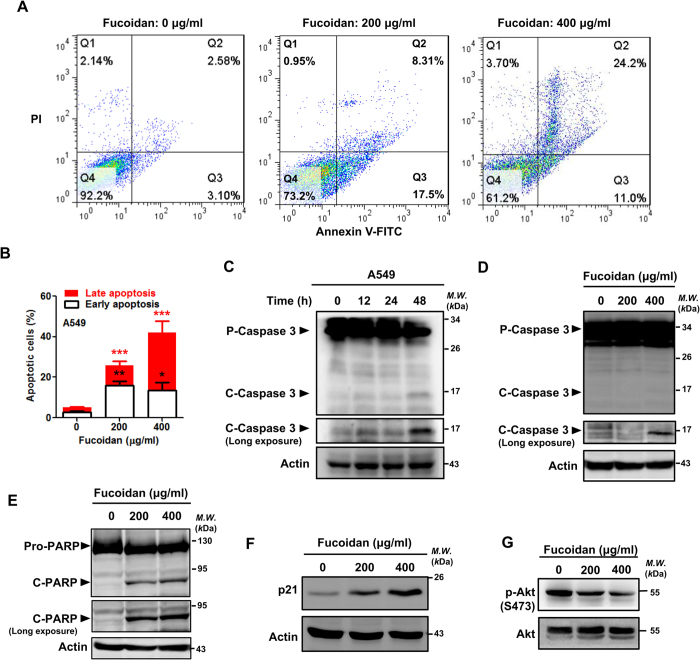
Fucoidan inhibits proliferation and induces apoptosis in A549 cells. (**A**) A549 cells were treated with fucoidan (200 and 400 μg/ml) for 48 h. The cells were stained with annexin V-FITC and PI and subsequently analyzed using flow cytometry. (**B**) Quantification of fucoidan-induced apoptotic cells by annexin/PI double-staining. Apoptosis was evaluated by determining the percentage of annexin V-positive cells. The data are representative of three independent experiments and are presented as the mean ± the SD, and error bars indicate the SD. Significant differences are shown (***P < 0.001, compared with the control group). (**C**) A549 cells were treated with fucoidan (200 μg/ml) for 12, 24 and 48 h. Then, Western blotting analysis of whole-cell lysates was performed to detect the expressions of pro (p)-caspase 3 and cleaved (c)-caspase 3. (**D**) A549 cells were treated with fucoidan (0–400 μg/ml) for 24 h, and Western blotting of whole-cell lysates was subsequently performed to detect Caspase 3 expression. (**E**) A549 cells were treated with fucoidan (0–400 μg/ml) for 48 h, and Western blotting of whole-cell lysates was subsequently performed to detect PARP expression. Actin was used as an internal control. (**F**) A549 cells were treated with fucoidan (200 and 400 μg/ml) for 24 h, and Western blotting analyses of whole-cell lysates were subsequently performed to detect p21 expression. (**G**) A549 cells were treated with fucoidan (0 to 400 μg/ml) for 24 h, and Western blotting analyses of whole-cell lysates were subsequently performed to detect p-Akt (Ser 473) expression. Total AKT was used as an internal control.

Using an MTT assay, we previously showed that fucoidan significantly inhibits the proliferation and viability of human lung cancer cells in dose- and time-dependent manners^7^. We found and applied that concentrations of fucoidan to A549 cells and CL1-5 cells are about 400–800 μg/ml and 100–200 μg/ml, respectively in our experiments. Next, using FACS analysis with propidium iodide (PI)/annexin V-FITC double staining, we investigated whether fucoidan induced apoptosis in A549 and CL1-5 lung cancer cells. As shown in Fig. 3A,B and Supplementary Fig. 1A,B, following 48 h of fucoidan treatment, apoptotic cells increased from 5% to 40–60% and from 3% to 18–50% in the A549 and CL1-5 cell lines, respectively. Furthermore, using Western blot analysis of the caspase activity, we demonstrated that fucoidan induced apoptosis in A549 and CL1-5 cells as indicated by the dose- and time-dependent expressions of the pro-apoptotic proteins caspase 3 and poly ADP ribose polymerase (PARP; Fig. 3C–E and Supplementary Fig. 1C). Moreover, using the Western blot assay and FACS analysis, we also found that fucoidan activated caspase 3 and PARP, leading to induction of apoptosis in LLC-1 cells (Supplementary Fig. 2).

Additionally, we also analyzed the effect of fucoidan on cell cycle distribution in lung cancer cells. Compared to the control cells, fucoidan treatment (200 μg/ml, 48 h) increased the proportions of A549 cells in sub-G1 and G1 phases from 1% to 4% and from 63% to 85%, respectively (Supplementary Fig. 3), and these results are comparable to those of another report^27^ that assessed the biological activity of low-dose fucoidan. Moreover, we confirmed that fucoidan up-regulated the expressions of cell cycle-related proteins, including p21, in both A549 and CL1-5 cells (Fig. 3F and Supplementary Fig. 1D). In contrast, fucoidan inhibited AKT phosphorylation in the treated cells (Fig. 3G and Supplementary Fig. 1E).

### Fucoidan induces ER stress-related signaling molecules in lung cancer cells

**Figure 4:**
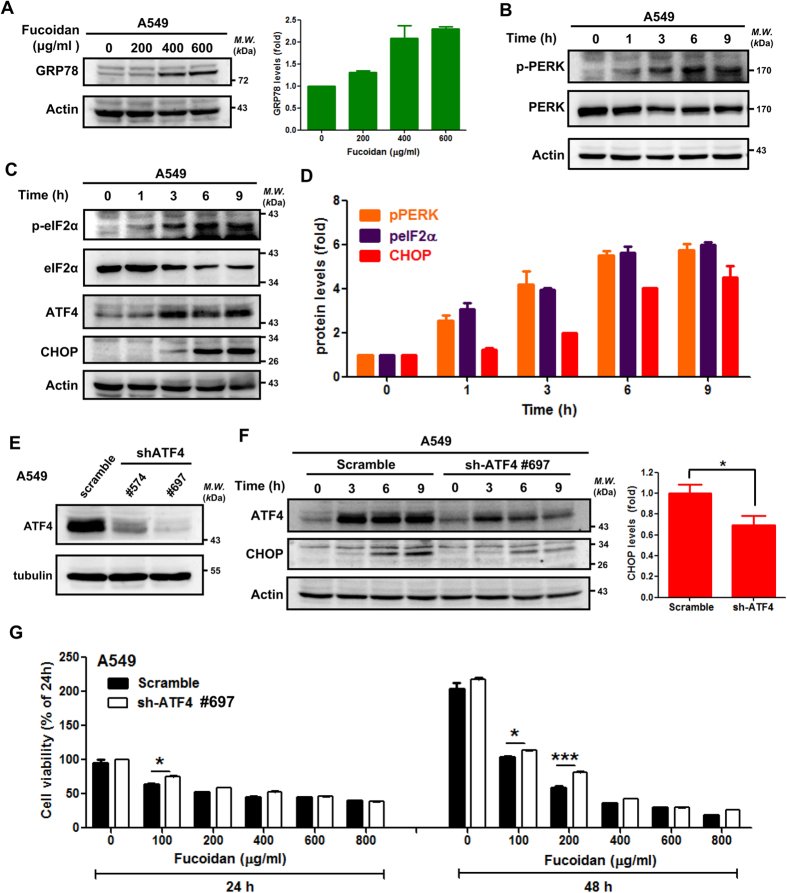
Fucoidan induces ER stress, and ATF4-shRNA (sh-ATF4) interferes with fucoidan-induced CHOP expression in lung cancer cells. (**A**) A549 cells were treated with fucoidan (0–600 μg/ml) for 48 h, and Western blotting analyses of whole cell lysates were subsequently performed to detect GRP78 expression. Right panel: Quantification of the intensities of GRP78 bands, which is representative of three separate determinations with ImageJ. (**B**,**C**) A549 cells were treated with fucoidan (400 μg/ml) for 1–9 h, and Western blotting analyses of whole-cell lysates were subsequently performed to detect the expressions of ER stress-related proteins. (**D**) Quantification of the band intensities of pPERK, peIF2α and CHOP (B-C) is representative of three separate determinations with ImageJ. (**E**) ATF4 expression in A549 cells was detected by Western blotting of whole-cell lysates following TG treatment for 24 h. (**F**) A549 cells (scramble and ATF4-knockdown) were incubated with fucoidan (200 μg/ml) for 0–9 h, and Western blotting of whole-cell lysates was subsequently performed to detect ATF4 and CHOP. Actin was used as an internal control. Right panel: The densitometric values of CHOP normalized to the relative actin value. Quantification of the intensities of CHOP bands was performed following fucoidan treatment for 9 h. The data are presented as the mean ± the SD, and error bars indicate the SD. Significant differences are noted (*P < 0.05 compared with the control group). (**G**) A549 cells (scramble and ATF4-knockdown cells) were treated with various doses of fucoidan (μg/ml) for 24 and 48 h. Cell viabilities were determined by crystal violet staining assays. Each group with fucoidan was normalized against each untreated control.

Following treatment with fucoidan, GRP78 expression was increased in A549 and CL1-5 cells (Fig. 4A and Supplementary Fig. 4A), which suggests that GRP78 is a gatekeeper in fucoidan-mediated activation of ER stress as previously reported^28^. Fucoidan also induced phosphorylation of the ER membrane protein PERK in a time-dependent manner (Fig. 4B and Supplementary Fig. 4B). We systematically analyzed molecules downstream of PERK. As shown in Fig. 4C and D, fucoidan induced eIF2α phosphorylation, and ATF4 and CHOP (i.e., molecules downstream of eIF2α) expressions were increased in a time-dependent manner in these cells. CHOP expression increased in CL1-5 cells treated with fucoidan for 24 h (Supplementary Fig. 4C). These results indicate the following: (i) fucoidan induced ER stress via activation of the PERK/eIF2α/ATF4/CHOP axes, which is consistent with other reports on ER stress; and (ii) activations of these axes are related to apoptosis in some cancer cells and involved in inhibition of cell viability^22^.

### Effect of ATF4 shRNAs on CHOP induction in fucoidan-induced ER stress in lung cancer

To further confirm the role of ATF4 in ER stress-mediated cell death, we performed ATF4-knockdown experiments using ATF4 shRNAs #574 (sh-S1) and #697 (sh-S2) in A549 cells. Based on the Western blotting analysis, we found that ATF4 knockdown reduced ER stress-induced ATF4 expression (Fig. 4E). In addition, CHOP, a downstream target of ATF4, is a marker of ER stress that promotes apoptotic cell death^15^. By examining the ER stress-induced activation of the ATF4/CHOP axis, we found that ATF4 knockdown (by ATF4 shRNA#697) abolished fucoidan-induced ATF4-mediated CHOP expression (Fig. 4F), which suggests that ATF4 plays an important upstream role in ER stress-mediated CHOP in the presence of fucoidan. To further investigate the role of ATF4 in lung cancer cell viability following fucoidan treatment, we performed a crystal violet staining assay to examine cell viability and proliferation. As illustrated in Fig. 4G, fucoidan effectively inhibited cell viability and proliferation in dose- and time-dependent manners. In contrast, abolishing ATF4 expression via ATF4 shRNA (#697) partially rescued the viability of low-dosage fucoidan-treated A549 cells although we also observed a reduction in high-dose of fucoidan treatment. Together, these results indicate that ATF4 may play a fucoidan-mediated cell death molecule that is involved in ER stress in lung cancer.

### Effect of CHOP knockdown on fucoidan-mediated ER stress in A549 cells

**Figure 5:**
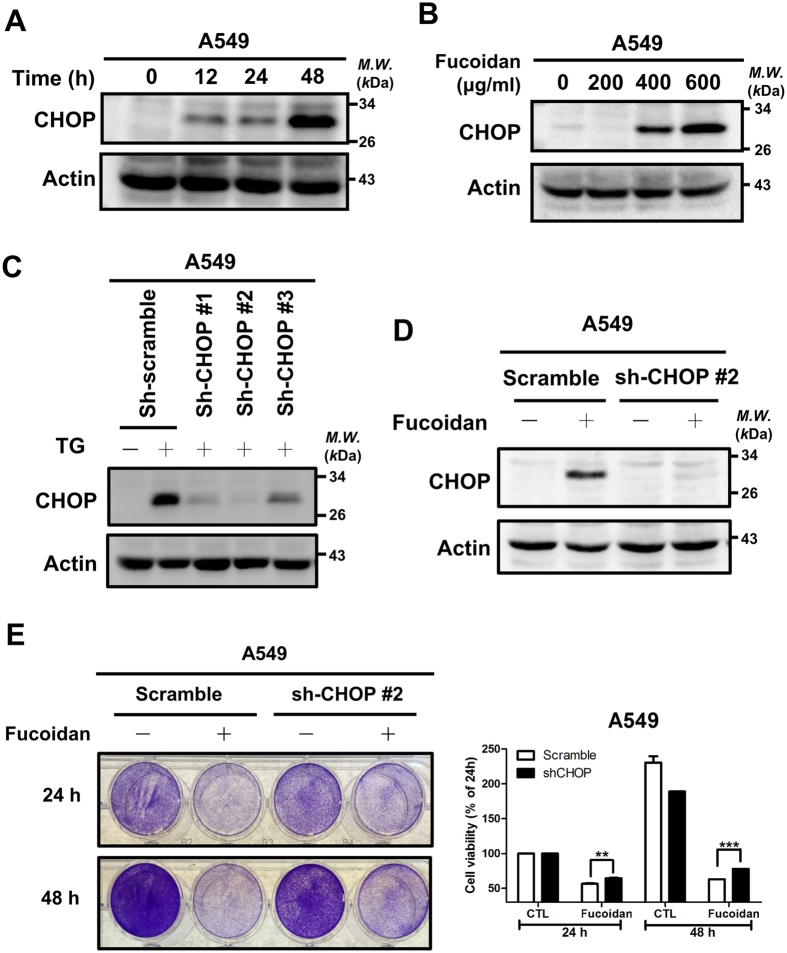
CHOP plays a role in fucoidan-inhibited cell viability. (**A**) A549 cells were treated with fucoidan (400 μg/ml) for 0 to 48 h, and Western blotting analysis of whole-cell lysates was subsequently performed to detect CHOP expression. (**B**) A549 cells were treated with fucoidan (0 to 600 μg/ml) for 6 h, and Western blotting analysis of the CHOP expression in whole-cell lysates was subsequently performed. (**C**) HEK293T cells were transfected with three types of sh-CHOP plasmid (sh-#1, sh-#2 and sh-#3) to produce lentiviruses as described in the Materials and Methods section. After the media were harvested, viruses containing individual CHOP-shRNAs were used to infect A549 cells for 24 h followed by puromycin selection. CHOP expression was detected by Western blotting of whole cell lysates following TG (50 nM) treatment for 6 h. (**D**) A549 cells (scramble and CHOP-knockdown) were incubated with fucoidan (200 μg/ml) for 12 h, and Western blotting of whole-cell lysates was subsequently performed to detect CHOP expression. Actin was used as an internal control. (**E**) A549 cells (scramble and CHOP-knockdown cells) were treated with fucoidan (200 μg/ml) for 24 and 48 h. Cell viabilities were determined by crystal violet staining assays. Each group of fucoidan-treated samples was normalized against each untreated control. The data are representative of three separate experiments and are presented as the mean ± the SD. Error bars indicate the SD. Significant differences are noted (**P < 0.01 and ***P < 0.001, compared with the control group).

CHOP expression is up-regulated upon ER stress, resulting in apoptosis^16^. To further explore and confirm the role of CHOP in fucoidan-induced apoptosis, we demonstrated that long-term treatment with fucoidan induces CHOP expression in time- and dose-dependent manners (Fig. 5A,B and Supplementary Fig. 5A,B). Using CHOP shRNAs, including #328 (sh-S1), #393(sh-S2) and #985 (sh-S3), we performed CHOP-knockdown experiments in A549 and CL1-5 cells. Based on Western blotting analyses, we used the ER stress inducer thapsigargin (TG) and found that CHOP knockdown led to a reduction in TG-induced ER stress-mediated CHOP expression (Fig. 5C and Supplementary Fig. 5C). Next, we demonstrated that CHOP knockdown (sh-S2) substantially inhibited fucoidan-mediated CHOP expression in A549 and CL1-5 cells (Fig. 5D and Supplementary Fig. 5D). Furthermore, CHOP knockdown rescued the fucoidan-inhibited cell viability in A549 cells (Fig. 5E). These results indicate that CHOP plays an essential role in fucoidan-induced ER stress-mediated apoptosis in lung cancer cells.

### The putative roles of Toll-like receptor 4 (TLR4) in fucoidan-induced intracellular generation of ROS and ER stress-mediated CHOP expression in lung cancer

**Figure 6:**
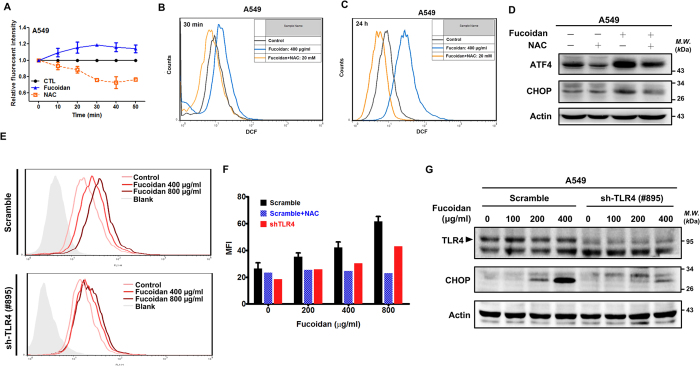
Fucoidan induces intracellular ROS generation in A549 cells. (**A**) A549 cells (5 × 10^4^ cells per well in 96-well plates) were treated with fucoidan (400 μg/ml) or NAC (5 mM) for 0–60 min and then stained with fluorescent dye (H_2_DCFHDA, DCF) for 30 min. The fluorescence intensity of DCF was detected using Infinite 200 PRO multimode microplate readers (TECAN, Switzerland) at excitation and emission wavelengths of 485 and 535 nm, respectively. (**B**,**C**) A549 cells were pre-treated with NAC (20 mM) for 30 min, followed by additional incubation with fucoidan (400 μg/ml) for 30 min (**B**) and 24 h (**C**). The fluorescence intensity of DCF was detected by flow cytometry. (**D**) A549 cells were pre-treated with 5 mM of NAC for 1 h and were then treated with fucoidan (400 μg/ml) for 24 h, and Western blotting analyses of whole-cell lysates were subsequently performed to measure ATF4 and CHOP expressions. (**E**) A549 cells (scramble and sh-TLR4) were treated with fucoidan (400 to 1600 μg/ml) or NAC (20 mM) for 30 min. The fluorescence intensity of DCF was detected by flow cytometry. (**F**) Quantification of MFI in an experiment (E) is representative of three separate determinations by FlowJo. (**G**) A549 cells (scramble and TLR4-knockdown cells) were treated with fucoidan (0 to 400 μg/ml) for 24 h. Cell viabilities were determined with crystal violet staining assays. Western blot analysis was performed to determine the TLR4 and CHOP protein levels.

Induction of intracellular ROS generation can trigger cell apoptosis via the ER stress pathway^29^. We previously found that fucoidan induces ROS and ER stress in lung cancer cells. However, the effect of fucoidan-induced ROS on ER stress in lung cancer cells is not fully understood. Here, using H_2_DCFDA, we measured the generation of intracellular ROS in fucoidan-treated lung cancer cells. As illustrated in Fig. 6A, following treatment of the cells with fucoidan, we observed a time-dependent increase in DCF fluorescence, which indicated that fucoidan induced intracellular ROS generation in A549 cells. Additionally, using flow cytometric assays, we confirmed that ROS generation in A549 cells was detectable after short-term (30 min) and long-term (24 h) fucoidan treatment (Fig. 6B and C), which indicates that fucoidan effectively induced ROS generation over a broad range of times. Alternatively, using the ROS inhibitor *N*-acetyl-l-cysteine (NAC) to block intracellular ROS generation, we demonstrated that fucoidan-induced ROS generation is abolished by pretreatment of cells with NAC (Fig. 6B and C). To further determine whether ROS participates in fucoidan-induced ER stress and its relevant bio-functions, we examined ATF4 and CHOP expression during co-treatment with fucoidan and NAC. ATF4 and CHOP expressions in A549 cells decreased following co-treatment with fucoidan and NAC compared with those in cells that were treated with fucoidan alone (Fig. 6D). These results indicate that fucoidan-induced ROS generation is involved in ER stress and ROS-stimulated ER stress-related molecule (ATF4 and CHOP) expression.

We previously demonstrated that lipopolysaccharide (LPS) binds to TLR and stimulates ROS, which results in expression of interleukin-1^30^. Moreover, fucoidan was shown to interact with TLR4 and activate the transcription nuclear factor kappa B (NF-κB) in embryonic human kidney epithelial cells^31^. Based on our previous studies, we hypothesized that TLR4 is involved in fucoidan-induced ROS. TLR4 knockdown experiments were conducted. We found that knockdown of TLR4 expression (shTLR4) abolishes fucoidan-induced ROS compared with that of the “scrambled” TLR4 control group (Fig. 6E). Second, we found that knockdown of TLR4 expression abolishes fucoidan-induced ROS in a dose-dependent manner (Fig. 6E). Third, we analyzed the mean fluorescence intensity (MFI) of fluorescent dyes (H_2_DCFHDA and DCF) and observed a 40% reduction of ROS in TLR4 knockdown cells upon fucoidan treatment (Fig. 6F). NAC effectively inhibited fucoidan-induced intracellular ROS generation in the “scrambled” TLR4 control group at various concentrations of fucoidan treatment compared with that of the TLR4 knockdown group (Fig. 6F), suggesting that the TLR4-dependent pathway is involved in fucoidan-induced ROS generation. The putative role of TLR4 in fucoidan-induced ER stress-mediated CHOP expression was also examined. Fucoidan induced CHOP expression in the “scrambled” TLR4 A549 control group in a dose-dependent manner (Fig. 6G). In contrast, fucoidan-induced CHOP expression was significantly inhibited in the TLR4-knockdown (sh-TLR4#895) A549 group (Fig. 6G). Together, our results demonstrate that TLR4 plays a partial role in fucoidan-induced ROS, which then promote ER stress and stress-mediated apoptotic gene expression.

## Discussion

Our previous findings show that orally administered fucoidan significantly reduces tumor volume/weight and TGFR protein levels in lung cancer LLC1-xenograft mice *in vivo*^6^,^7^; in our current study, we reported that pre-treatment of LLC1-xenograft mice with fucoidan could prevent and reduce tumor proliferation in mice (Fig. 1). In brief, we demonstrated that continuous oral feeding with fucoidan significantly reduces tumor volumes and induces ATF4 and CHOP protein expressions in LLC1-bearing mice *in vivo*. We found that fucoidan (i) effectively inhibits the proliferation of lung cancer cells via induction of G1 phase arrest and (ii) promotes cell death via apoptosis. One fucoidan-inducing apoptosis mechanism involves the induction of cell cycle arrest and enhancement of ROS levels via TLR4. We also found that fucoidan triggers ROS-mediated ER stress and activates UPR pathways to induce cell death (Supplementary Fig. 6). Taken together, our results demonstrate the pharmaceutical efficacy of fucoidan in a mouse lung cancer model. We also provide the first evidence of TLR4-mediated fucoidan induction of ROS that is dependent on the PERK/ATF4/CHOP axis and apoptosis as the underlying mechanisms.

The molecular mechanism of anticancer effects of fucoidan is summarized as the signaling pathways associated with the processes of apoptosis, cell cycle arrest, anti-angiogenesis, inhibiting metastasis (migration and invasion), etc. Our current finding proposed that fucoidan-induced apoptosis of lung cancer cell results from activation of the TLR4/ROS/ER stress axis and the mediation of downstream PERK-ATF4-CHOP signaling pathways. In addition, the fucoidan-mediated transducing signal pathways relevant to fucoidan-induced apoptosis have been reported as a pathway of the FAK-AKT axis^32^, activation of caspase-8 -9 and -3 pathways^33^, and suppression of the ASK1-p38 signaling pathway^34^. Our findings are that fucoidan induces apoptosis in lung cancer cells, as detected by expressions of the pro-apoptotic proteins caspase 3 and PARP, which is comparable to reports from other investigators^35^. On the other hand, fucoidan induces G1 arrest in cell cycle progression, leading to apoptosis in cancer cells^3^. In our study, we found that fucoidan treatment increases the proportions of lung cancer cells in G1 phases and the results are comparable to those of another reports^36^,^37^. Several transducing signal pathways involved in fucoidan-mediated cell cycle arrest have been proposed, such as fucoidan activation of the AKT signaling pathway^36^ and activation of p16INK4a-Rb and p14Arf-p53 pathways^37^.

Under ER stress, cells activate various signaling pathways to induce apoptosis and other reactions. ER stress–induced apoptosis is implicated in various pathological conditions, but the mechanisms linking ER stress–mediated signaling to downstream apoptotic pathways need to be studied^17^. CHOP is a marker of ER stress-mediated apoptosis; additionally, ATF4 has known pro-apoptotic functions that mediating the regulation of CHOP expression upon ER stress^15^. Here we found that fucoidan induces CHOP expression in lung cancer cells; and the PERK-eIF2α-ATF4-CHOP signaling pathway is one of major pathways in ER stress-mediated apoptosis^17^.

During tumor progression, elevated ROS promotes many tumor properties, including proliferation, transformation and metastasis^18^,^38^. Tumor cells also manipulate and increase multiple antioxidant proteins to reduce the toxicity of endogenous ROS. Thus, a delicate and complicated balance of “reduction” and “oxidation” (redox) modulation of intracellular ROS expression exists in cancer cells to maintain the malignant functions while eliminating ROS-induced toxicity. However, induction of excess ROS in cancer cells via therapeutic approaches, such as chemotherapy and radiotherapy, may trigger ROS-mediated cell death mechanisms^39^. ROS induce ER stress through modification of proteins and lipids^29^. Here, we examined effects of fucoidan on ROS-mediated ER stress and apoptosis, developing a potential therapeutic strategy for the treatment of lung cancer patients.

Under normal conditions, lung cancer cells keep a well-maintained redox balance. Following addition of fucoidan to testing cancer cells, there is a rapid increase in cellular ROS levels, disrupting the intracellular redox balance. The sudden fucoidan-induced redox dysfunction or ROS accumulation induces ER stress and activates apoptotic-related molecules. In addition, we found that fucoidan-induced ROS, as one of the upstream “initiating” signaling molecules, could be inhibited by the antioxidant NAC, followed by decreased ER stress and subsequent reduced activation of downstream apoptotic molecules and/or reactions in the cells. Importantly, we demonstrated that fucoidan induces ROS in a very short period (within 30 min). In contrast, fucoidan has been reported to promote mitochondrial-derived ROS generation after approximately 24–48 h^25^. Thus, the fucoidan-induced increase in ROS may occur at an early stage (receptor-mediated) or at a later stage (mitochondria-derived). Based on our current results from lung cancer cells, fucoidan can function as a redox modulation agent for treatment of lung cancer.

Recent findings indicate that the TLRs are expressed not only on immune cells, including macrophages^30^, but also on some cancer cells. In lung cancer cells, TLR4 facilitates tumor progression, metastasis and drug resistance^40^,^41^. Specifically, TLR4 activation triggers ROS generation^42^. It is unknown how fucoidan induces ROS generation in lung cancer cells. Thus, we hypothesized that TLR4 activation may be associated with fucoidan-induced ROS generation. Our results demonstrated that knockdown of TLR4 abolishes fucoidan-induced ROS levels and CHOP expression, indicating TLR4 involvement in fucoidan-induced ROS generation and ER stress in lung cancer. However, we could not exclude the possibility that the TLR4-shRNAs we used were inefficient at inhibiting endogenous TLR4 expression and function in our cells. It is also likely that other TLRs are involved in the interaction with fucoidan. Fucoidan increases ROS levels over a broad range of time, suggesting that fucoidan induces the activation of TLR4-mediated ROS generation. Alternatively, we could not exclude the possibility that the ROS-mediated mitochondrial pathway and apoptosis are involved in our system^23^,^24^.

We previously demonstrated that fucoidan effectively inhibits cell growth and migration via induction of Smurf2-dependent ubiquitin degradation of TGFβ (TGF) receptors (TGFR) in lung cancer cells^7^. Here we also found that fucoidan activation of TLR4 and TLR4-mediated ROS-induced ER stress (PERK-eIF2α-ATF4-CHOP pathway), which involved in fucoidan-induced apoptosis (activation of caspase 3 and PARP) of lung cancer cells. These findings suggest that there are multiple signaling pathways involved in fucoidan-mediated antitumor functions within lung cancer cells; however, the binding or targeting molecule or receptor of fucoidan on the surface of cancer cell membranes is still unclear. Interestingly, we previously reported that fucoidan may serve as a ligand to interact with TLR4 or scavenger receptor A on cellular surfaces of macrophages, resulting in induction of cytokine expression^43^,^44^,^45^. Alternatively, Tollip, a negative regulator of TLR4, cooperates with Smad7 to mediate ubiquitination-dependent degradation of TGFR^46^. More recently, we found that fucose-containing polysaccharides from Reishi enhances the cooperation of Tollip with the TGFR/Smurf2/Smad7 complex and further modulates the degradation of TGFR^47^. Thus, it would be an interesting topic of research for whether there is any association between degradation of TGFR and activation of TLR4 upon fucoidan treatment.

Recently, the interplay between TGF/TGFR and ROS-mediated oxidative stress has been discussed. Evidence indicates that TGF/TGFR is able to control ROS production directly or by modulating anti-oxidation systems. Several studies have shown that ligation of TGF to TGFR can induce ROS production in different cellular compartments, including that TGF-*β* induces ROS production in mitochondria^39^. By contrast, ROS can influence TGF/TGFR signaling and increase TGF expression as well as its activation from the latent complex. It is demonstrated that ROS mediates the TGF/TGFR-regulated expression of a group of genes, but little is known about how ROS may regulate the activation of TGF/TGFR-mediated intracellular signal transduction. It has been reported that Smad2-mediated signaling seems to be sensitive to ROS effects, due to studies which showed that TGF-stimulated Smad2 phosphorylation can be inhibited by N-acetyl cysteine^39^. Elucidating the complex interplay and roles of TGF/TGFR-mediated signalings and ROS-induced signal redox stress in cancer is important for the understanding of their participation in tumorigenesis. In the future, we will examine the possibility of TLR4 activation and induced ROS involved in fucoidan-induced degradation of TGFRs and the downstream signaling pathways.

It is reported that fucoidan, polysaccharides derived from various brown seaweeds, exert different anti-cancer effects. However, there are many “factors” of fucoidan involved in anti-cancer functions; for example, sources of fucoidan (species of brown seaweeds)^23^,^24^,^25^, effective concentration, structural characteristics, sulfate content, molecular weight, purity, isolation/extraction methods, etc., as well as importantly the kind of cancer cells to be tested. Here, using HiQ-fucoidan from *Laminaria japonica*, we found that fucoidan also prevents and inhibits lung tumor growth at higher concentrations (Supplementary Fig. 7) *in vitro* and *in vivo*, even though the species from which HiQ-fucoidan (*Laminaria japonica*) and fucoidan (*Fucus vesiculosus*) are derived from differ. Under the same experimental conditions, we found that both types of fucoidan exert similar biological functions in cellular apoptosis and anti-lung cancer activity. In our future work, we will further examine and compare the relationship between structural characteristics and anti-cancer activity within HiQ-fucoidan and fucoidan. Overall, our current results suggest that fucoidan exerts both preventive and therapeutic effects in a lung cancer mouse model. Therefore, we propose using fucoidan as a nutritional/dietary supplement to prevent and inhibit lung cancer progression.

## Materials and Methods

### Cell lines and cell culture

Human non-small cell lung adenocarcinoma cell lines A549 and CL1-5 were obtained from Dr. P.-C. Yang (NTU, Taiwan). The human embryonic kidney (HEK) 293 T cell line was obtained from Dr. I.-T. Chen (NYMU, Taiwan). The mouse Lewis lung carcinoma LLC1 was purchased from the Bioresource Collection and Research Center (BCRC, Hsinchu, Taiwan). Cells were cultured as previously described^7^,^48^.

### Reagents and antibodies

Fucoidan from *Fucus vesiculosus* was obtained from Sigma-Aldrich Co. (St. Louis, MO, USA) as fucoidan. Fucoidan from *Laminaria japonica* was a gift from Hi-Q Marine Biotech International, Ltd. (Taiwan) as HiQ-fucoidan. PI, NAC, anti-actin (AC-74), anti-pERK1/2 (MAPK-YT) and anti-p21 (CP74) were purchased from Sigma-Aldrich Co. (St. Louis, MO, USA). Lipofectamine 2000 was purchased from Invitrogen (Grand Island, NY, USA). Anti-AKT, anti-p-AKT (S473) and anti-mouse IgG-HRP were purchased from Santa Cruz Biotechnology (CA, USA). Anti-CHOP, anti-GRP78, anti-eIF2α, and anti-rabbit IgG-HRP were purchased from GeneTex, Inc. (Hsinchu, Taiwan). Anti-caspase3 (8G10), anti-p-PERK (T980; 16F8), anti-PERK (D11A8), anti-p-eIF2α (Ser51, 119A11), anti-TLR4 (D8L5W) and anti-ATF4 (D4B8) were purchased from Cell Signaling (Beverly, MA, USA).

### Western blot analysis

Cancer cells from each experimental condition were rinsed with cold phosphate-buffered saline (PBS) containing 1% Na_3_VO_4_ and harvested by scraping into lysis buffer (10 mM HEPES pH 7.9, 10 mM KCl, 0.1 mM EDTA, 0.1 mM EGTA, proteinase inhibitors). Whole-cell lysates were centrifuged at 13,000 × *g* for 10 min at 4 °C. Protein concentration of the supernatant was determined by Bradford assays (Bio-Rad, Hercules, CA, USA), and bovine serum albumin (BSA; Thermo Fisher Scientific, Rockwood, TN) was used as a standard. Cell extracts (40 μg) were subjected to Western blot analysis. Actin expression was used as an internal control. The detailed procedure has been described previously^6^. Quantification of the detected protein band intensities was determined using ImageJ (National Institute of Mental Health, Bethesda, MD, USA) and is representative of three separate experiments.

### Cell viability assay via crystal violet staining

Cells (1 × 10^5^ cells per well) were seeded in a 12-well plates and incubated overnight prior to treatment with fucoidan for 48 h. After incubation, each well was rinsed with PBS. The attached cells were fixed and stained with 1% crystal violet (Bioman, Taiwan) solution in 30% ethanol (PanReac AppliChem, USA) for 30 min followed by staining with 33% acetic acid (Bioman, Taiwan) to dissolve the crystal violet. Cell viability was determined by detecting absorbance wavelengths from 570 to 670 nm.

### Apoptosis analysis

Cells (5 × 10^5^ cells per plate) were seeded in a 6-cm plate and incubated overnight prior to treatment with fucoidan (100 to 400 μg/ml) for 48 h. These cells were harvested by trypsinization and washed with 1.0 ml cold PBS. Fucoidan-induced apoptosis was detected using an Alexa Fluor 488 Annexin V Apoptosis Kit (Life Technologies).

### Detection of intracellular ROS

ROS was measured using 2′,7′-dichlorodihydrofluorescein diacetate (H_2_DCFDA, Life Technologies) as previously reported^30^. Cells (1 × 10^4^ cells per well) were seeded in triplicate in a 96-well plate and incubated overnight prior to treatment with fucoidan (20 to 400 μg/ml) for 10 to 60 min. After the cells were washed with PBS, they were stained with 200 μM H_2_DCFDA and incubated at 37 °C for 30 min. After incubation, cells were washed with PBS. The medium was replaced with fresh cell medium, and the cells were incubated at 37 °C for 30 min. Fluorescence intensity of the DCF probe was detected using Infinite 200 PRO multimode microplate readers (TECAN, Switzerland) or FACScan flow cytometry (Becton Dickinson, Germany) at excitation and emission wavelengths of 485 and 535 nm, respectively.

### *In vivo* lung cancer mouse model

Male C57BL/6 strains of mice (aged 6 to 8 weeks) were obtained from the National Laboratory Animal Center of Taiwan. Mice were raised under pathogen-free conditions in the Animal Center of National Yang-Ming University (NYMU). All experimental methods involving animals were carried out in accordance with guidelines and regulations of the Institutional Animal Care and Use Committee (IACUC) of NYMU, and all experimental protocols were approved by the IACUC of NYMU.

To examine the effect of fucoidan on tumor volume, the mice were randomly distributed into two groups and orally fed with either ddH_2_O (control group) or fucoidan at 24 mg/kg/day (experimental group). After oral feeding with fucoidan for 2 weeks, each mouse was inoculated with LLC1 cells (2 × 10^5^ cells per mouse) into the hypodermic dorsum and orally fed with fucoidan for 3 weeks. The detailed procedure has been described previously^7^.

### RNA interference with sh-ATF4, sh-CHOP or sh-TLR4

Specific shRNA oligonucleotides targeting ATF4, CHOP and TLR4 based on the human *ATF4* (NM_001675.2), *DDIT3* (NM_004083), and *TLR4* genes (NM_138554.3), respectively, were obtained from the Taiwan National RNAi Core Facility Platform. The targeting sequences for ATF4, CHOP and TLR4 are listed in Supplementary Table S1, and the procedure has previously been described^7^.

### Statistical analysis

All data are expressed as the mean ± standard error. Significant differences between two groups were determined by *t*-test analyses using statistical software, *GraphPad Prism5*. A *P*-value of <0.05 was considered statistically significant (*P < 0.05, **P < 0.01 and ***P < 0.005).

## Additional Information

**How to cite this article:** Hsu, H.-Y. *et al*. Fucoidan induces Toll-like receptor 4-regulated reactive oxygen species and promotes endoplasmic reticulum stress-mediated apoptosis in lung cancer. *Sci. Rep.* **7**, 44990; doi: 10.1038/srep44990 (2017).

**Publisher's note:** Springer Nature remains neutral with regard to jurisdictional claims in published maps and institutional affiliations.

## Supplementary Material

Supplementary Data
