# Supplementary material for: Fucoidan induces Toll-like receptor 4-regulated reactive oxygen species and promotes endoplasmic reticulum stress-mediated apoptosis in lung cancer
Source: Sci Rep. 2017 Mar 23;7:44990. doi: 10.1038/srep44990 (PMC5362958; doi:10.1038/srep44990)
Supplement: Supplementary Data [file srep44990-s1.pdf]

Fucoidan induces Toll-like receptor4-regulated reactive oxygen species and promotes endoplasmic reticulum stress-mediated apoptosis in lung cancer

Hsien-Yeh Hsu, Tung-Yi Lin, Mei-Kuang Lu, Pei-Ju Leng, Shu-Ming Tsao, and Yu-Chung Wu

Supplementary information

Supplementary method

Cell cycle analysis

A549 Cells (5×10^5 cells per plate) were seeded in a 6-cm plate and incubated overnight before treating with fucoidan (200 μg/ml) for 24 h. The cells were harvested by trypsinization and were washed with 1.0 ml cold PBS. The cell pellets were centrifuged at 1800 ×g for 15 min and were incubated with 70% ethanol at -20°C for 30 min. After this treatment, the cells were centrifuged and re-suspended in PI master mixture buffer (PBS 50 μl, PI 100 μg/ml (Sigma-Aldrich Co.) and 10 μl RNase (Invitrogen)) at 37°C for 30 min followed by flow cytometry analysis (BD FACSCalibur). A minimum of 10,000 cells/sample were collected, and each DNA histogram was further analyzed by using FlowJo 7.6.1 software (FLOWJO, LLC., USA) for cell cycle analysis.

HiQ-Fucoidan

Fucoidan from *Laminaria japonica* was a gift from Hi-Q Marine Biotech International, Ltd. (Taiwan) as HiQ-fucoidan.

Supplementary Table I. Sequence of shRNA

shRNA	Target sequences (5' to 3')	shRNA sequence (5' to 3')
ATF4 #574 (shATF4)	CCACTCCAGATCATTCCTTTA	CCGGCCACTCCAGATCATTCCT TTACTCGAGTAAAGGAATGATC TGGAGTGGTTTTT
ATF4 #697 (shATF4)	ACCTTCTGACCACGTTGGATG	CCGGACCTTCTGACCACGT TGGATGCTCGAGCATCCAA CGTGGTCAGAAGGTTTTTTG
CHOP#328 (shCHOP#1)	CTGCACCAAGCATGAACAATT	CCGGCTGCACCAAGCATGA ACAATTCTCGAGAATTGTTC ATGCTTGGTGCAGTTTTTG
CHOP#393 (shCHOP#2)	TGAACGGCTCAAGCAGGAAAT	CCGGTGAACGGCTCAAGCA GGAAATCTCGAGATTTCCTG CTTGAGCCGTTCATTTTTG
CHOP#985 (shCHOP#3)	AGGTCCTGTCTTCAGATGAAA	CCGGAGGTCCTGTCTTCAGA TGAAACTCGAGTTTCATCTG AAGACAGGACCTTTTTTG
TLR4 #895 (shTLR4)	CGTTTGGTTCTGGGAGAATTT	CCGGCGTTTGGTTCTGGGAGAA TTTCTCGAGAAATTCTCCCAGA ACCAAACGTTTTTG

Supplementary Figure 1.

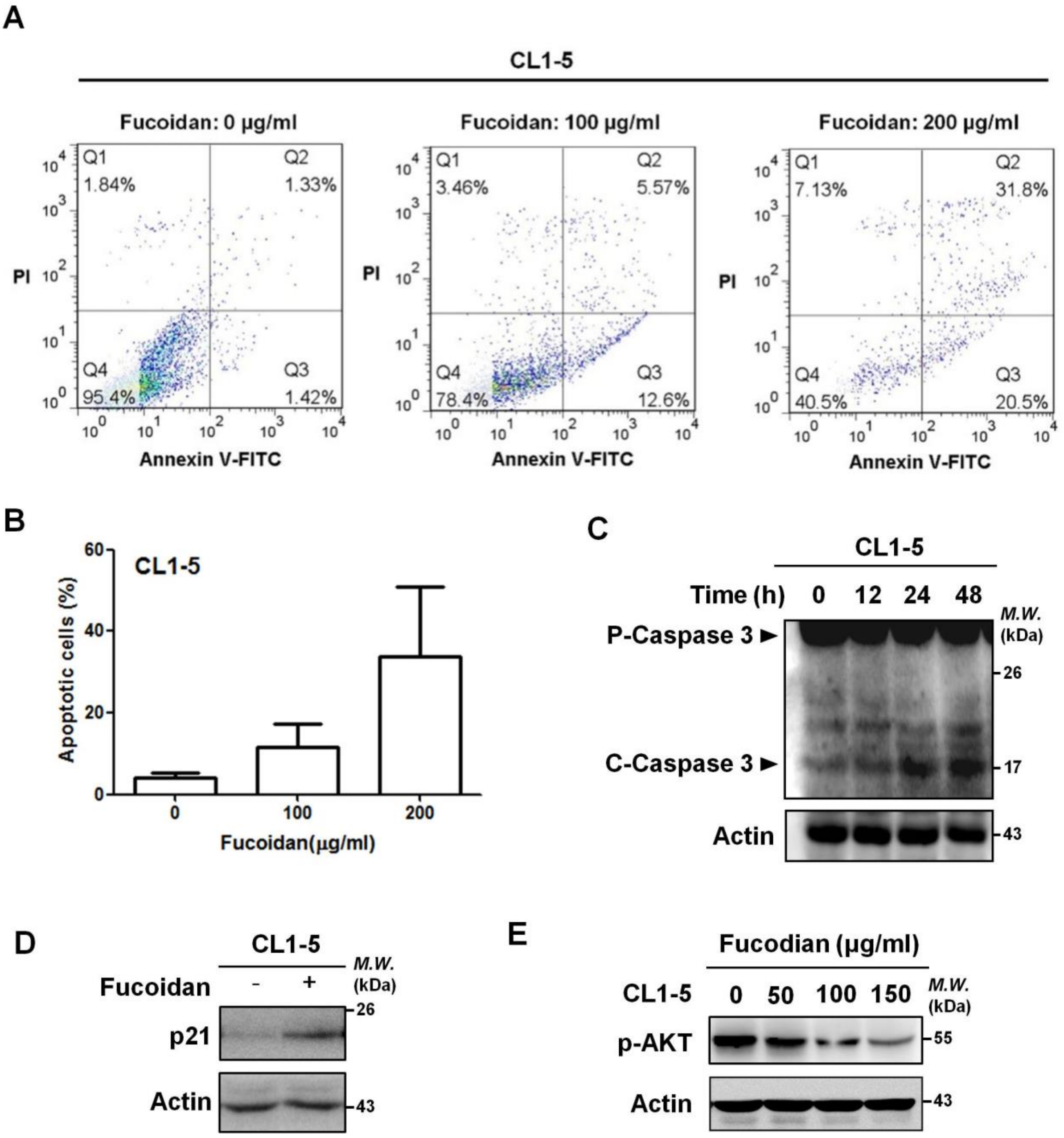

Supplementary Figure 1. Fucoidan induces apoptosis in CL1-5 cells.

(A) CL1-5 cells (5×10^5 cells) were treated with fucoidan (100 and 200 μg/ml) for 48 h. The cells were stained with annexin V-FITC and PI and subsequently detected with flow cytometry. **(B)** Quantification of the fucoidan-induced apoptotic cell death was performed by annexin V/PI double-staining. Apoptosis was evaluated by determining the percentages of annexin V-positive cells. The data are representative of three independent experiments and are presented as the mean ± the SD. Error bars indicate the SD. **(C)** CL1-5 cells (5×10^5 cells) were treated with fucoidan (100

μg/ml) for 12, 24 and 48 h. The treatments were followed by Western blotting analysis of whole cell lysates to detect pro (p)-caspase 3 and cleaved (c)-caspase 3 expression. **(D)** CL1-5 cells were treated with fucoidan (100 μg/ml) for 24 h, and Western blotting of whole cell lysates was subsequently performed to detect p21 expression. **(E)** CL1-5 cells were treated with fucoidan (0 to 150 μg/ml) for 24 h, and Western blotting analyses of whole-cell lysates were subsequently performed to detect p-Akt (Ser 473) expression. Actin was used as an internal control.

Supplementary Figure 2.

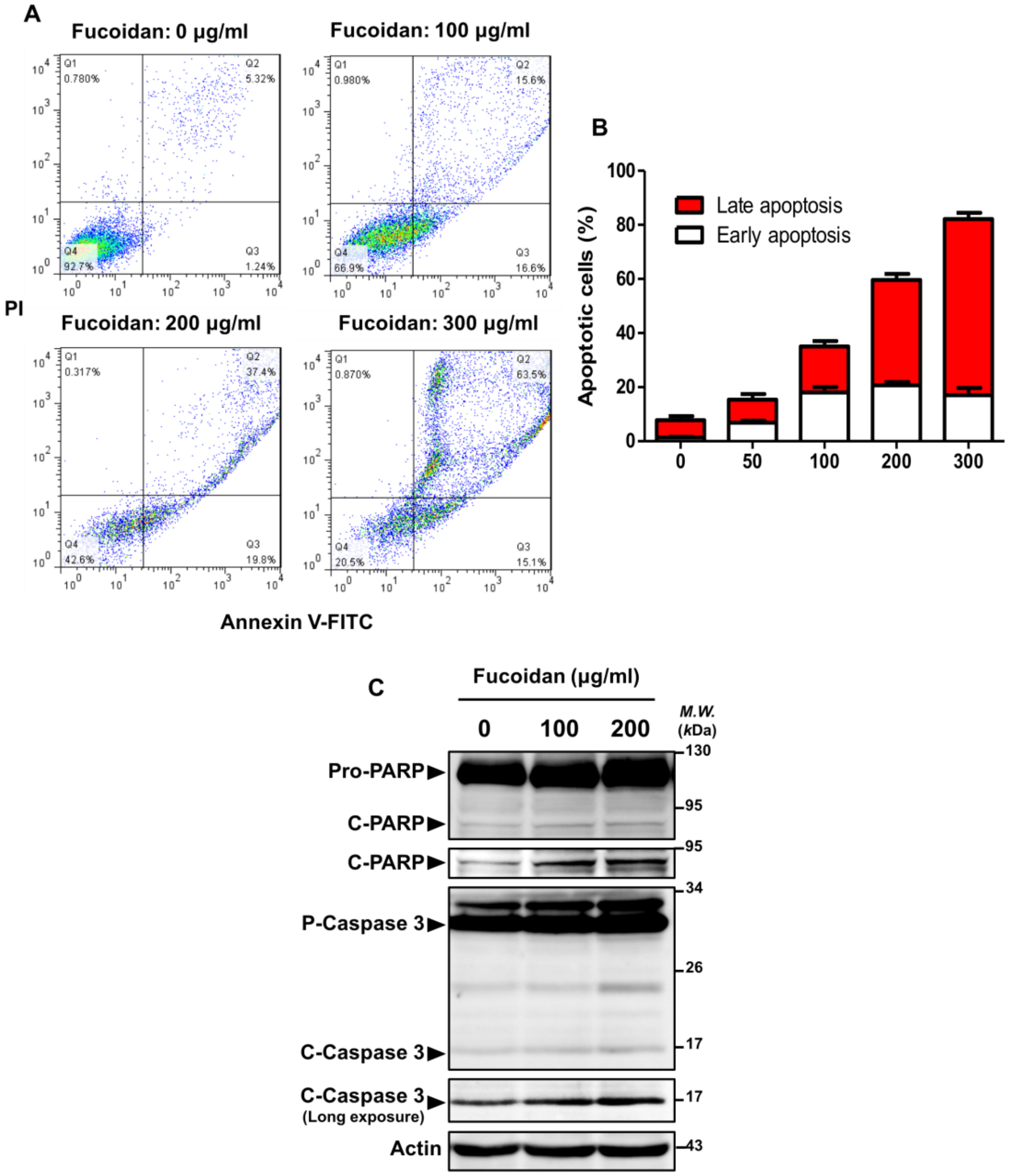

Supplementary Figure 2. Fucoidan induces apoptosis in LLC1 cells.

(A) LLC-1 cells were treated with fucoidan (0–300 μg/ml) for 48 h. The cells were stained with annexin V-FITC and PI and subsequently analyzed using flow cytometry. **(B)** Quantification of fucoidan-induced apoptotic cells by annexin/PI double-staining. Apoptosis was evaluated by determining the percentage of annexin V-positive cells. The data are representative of three independent experiments and are presented as the mean ± the SD, and error bars indicate the SD. **(C)** LLC-1 cells were treated with fucoidan (0–200 μg/ml) for 24 h, and Western blotting of whole-cell lysates was subsequently performed to detect PARP and Caspase 3 expression.

Supplementary Figure 3.

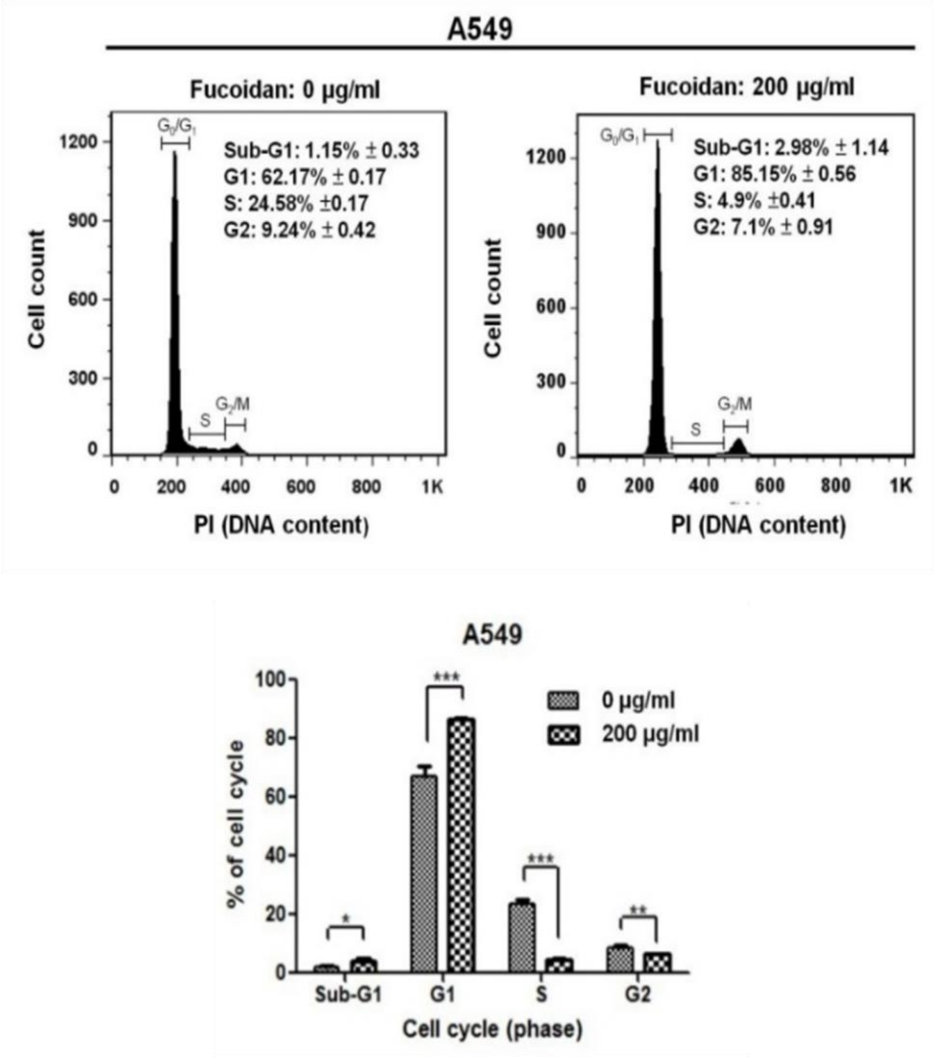

Supplementary Figure 3. Fucoidan induces cell cycle arrest in A549 cells.

A549 cells (5×10^5 cells) were treated with fucoidan (200 μg/ml) for 24 h. Then, the cells were collected, fixed, and stained with propidium iodide for flow cytometry analysis. The DNA content is represented by the x-axis, and the number of cells counted is represented by the y-axis. Statistically significant differences are shown (*$P < 0.05$, **$P < 0.01$, ***$P < 0.001$) compared with the control group.

Supplementary Figure 4.

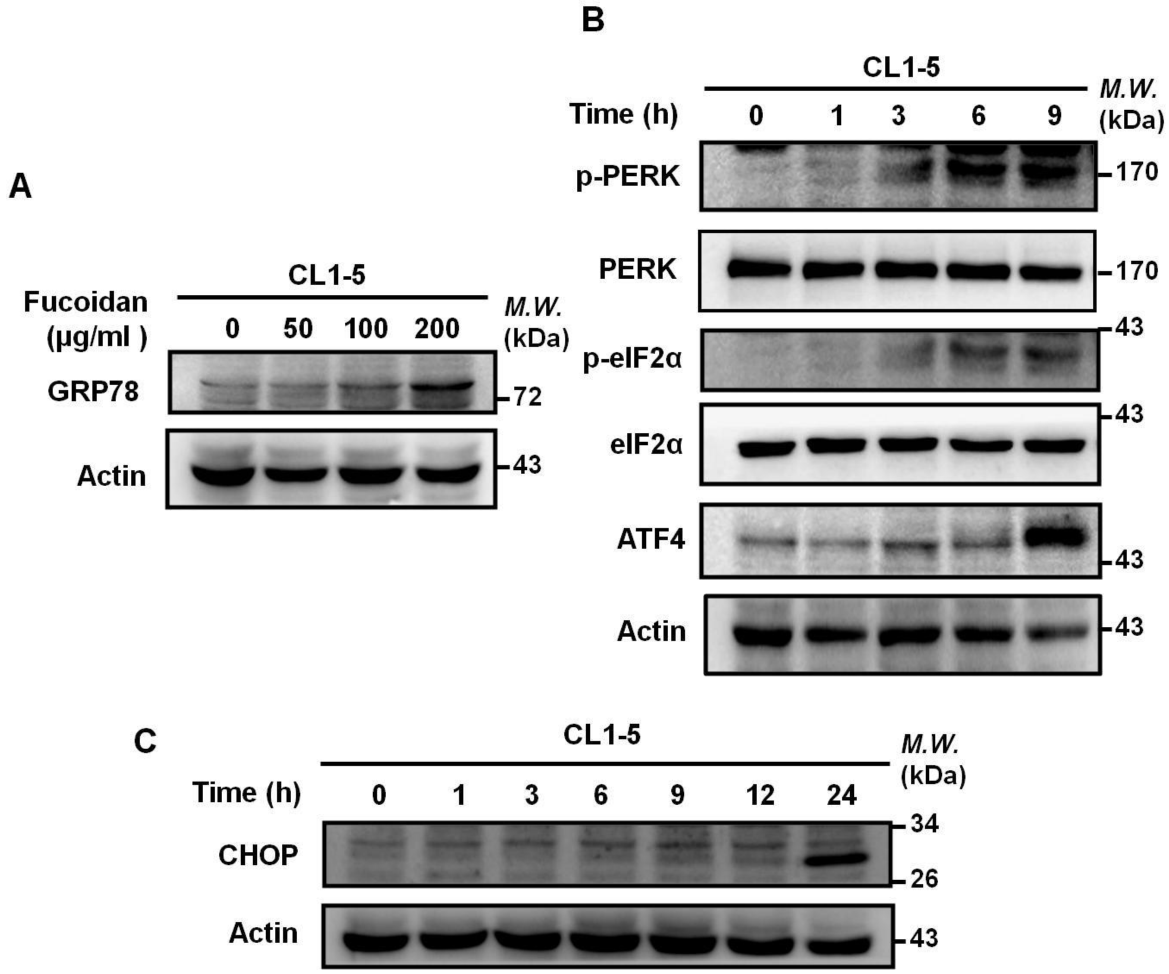

Supplementary Figure 4. Fucoidan induces ER stress in CL1-5 cells.

(A) CL1-5 cells (5×10^5 cells) were treated with fucoidan (0–200 μg/ml) for 48 h, and Western blotting analyses of whole-cell lysates were subsequently performed to detect GRP78 expression. **(B)** CL1-5 cells (5×10^5 cells) were treated with fucoidan (200 μg/ml) for 1, 3, 6 and 9 h, and Western blotting analyses of whole-cell lysates were subsequently performed to detect the expressions of p-PERK (Thr980), p-eIF2α (Ser51), and ATF4. PERK, eIF2α and actin were used as internal controls for loading. **(C)** CL1-5 cells (5×10^5 cells per 6 cm plate) were treated with fucoidan (200 μg/ml) for 0 to 24 h. The treatment was followed by Western blotting analysis of whole-cell lysates to detect CHOP expression.

Supplementary Figure 5.

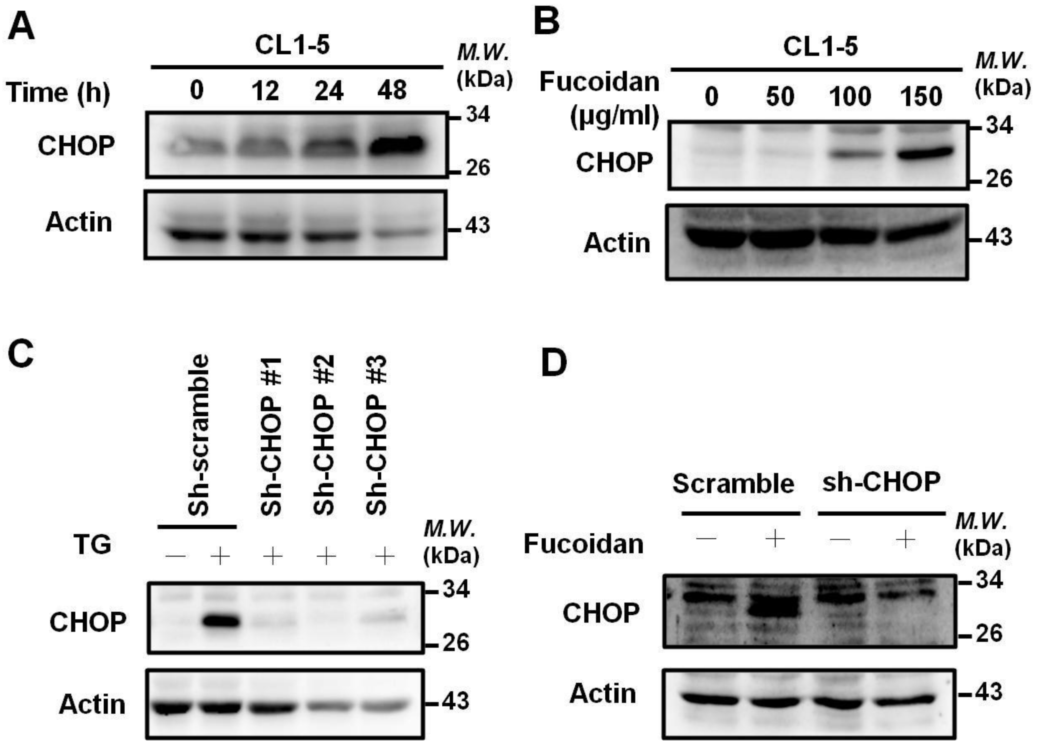

Supplementary Figure 5. Fucoidan induces CHOP expression in CL1-5 cells.
(A) CL1-5 cells (5×10^5 cells) were treated with fucoidan (200 μg/ml) for 0 to 48 h, and Western blotting analyses of whole-cell lysates were subsequently performed to detect CHOP expression. **(B)** CL1-5 cells (5×10^5 cells) were treated with fucoidan (0 to 150 μg/ml) for 24 h, and Western blotting analyses of CHOP expression in whole-cell lysates were subsequently performed. **(C)** HEK293T cells were transfected with three types of sh-CHOP plasmid (sh-#1, sh-#2 and sh-#3) to produce lentiviruses as described in the Materials and Methods section. After the media were harvested, viruses containing individual CHOP-shRNAs were used to infect CL1-5 cells for 24 h followed by puromycin selection. CHOP expression was detected by Western blotting of whole-cell lysates following thapsigargin (TG, 50 nM) treatment for 6 h. **(D)** CL1-5 cells (scramble and CHOP-knockdown, sh-CHOP #2) were incubated with fucoidan (200 μg/ml) for 12 h, and Western blotting of whole-cell lysates was subsequently performed to detect CHOP expression. Actin was used as an internal control.

Supplementary Figure 6.

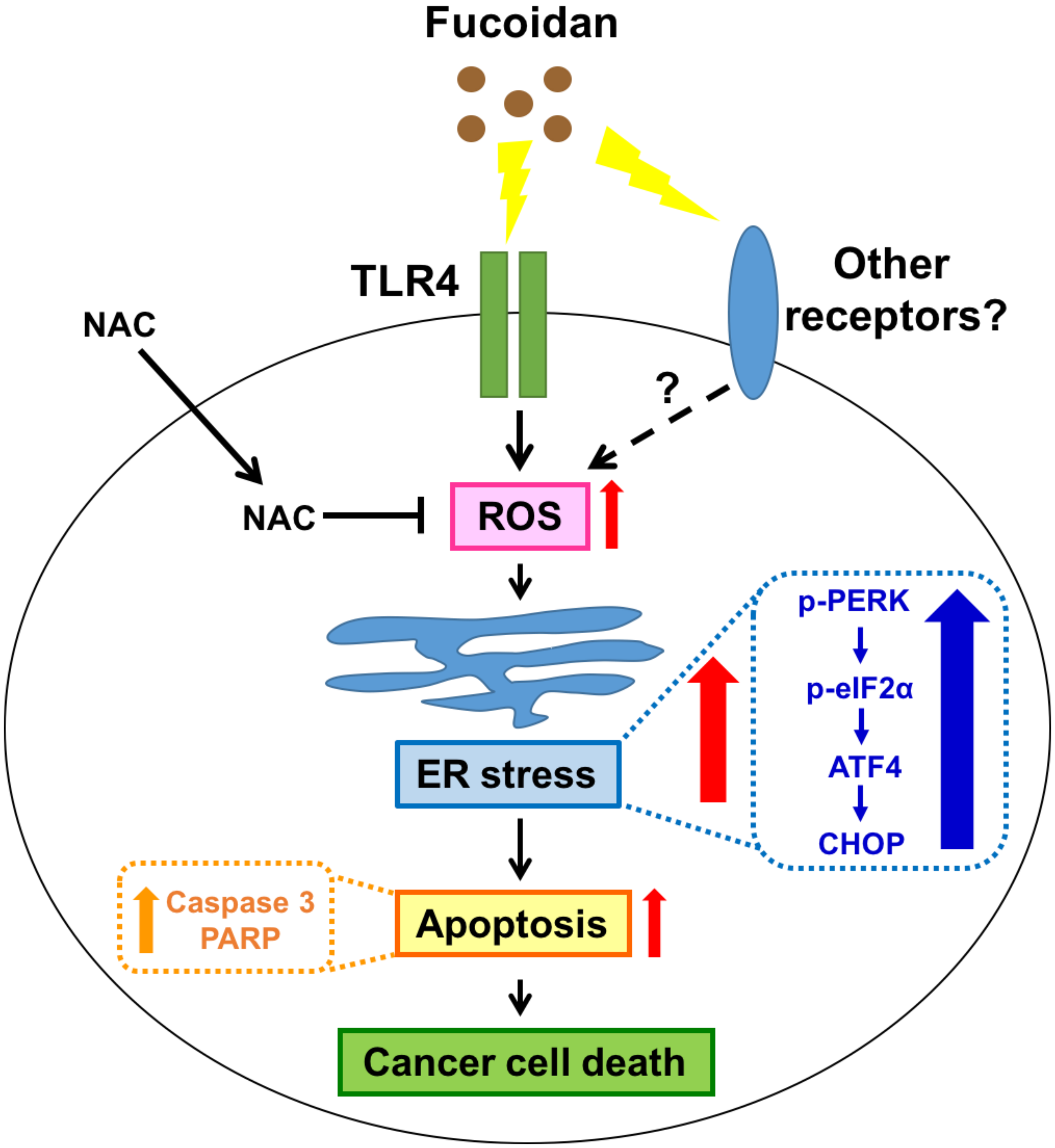

Supplementary Figure 6. Proposed model illustrating the roles of fucoidan related to TLR4-mediated induction of ROS-mediated ER stress-dependent apoptosis in lung cancer cells.

Supplementary Figure 7.

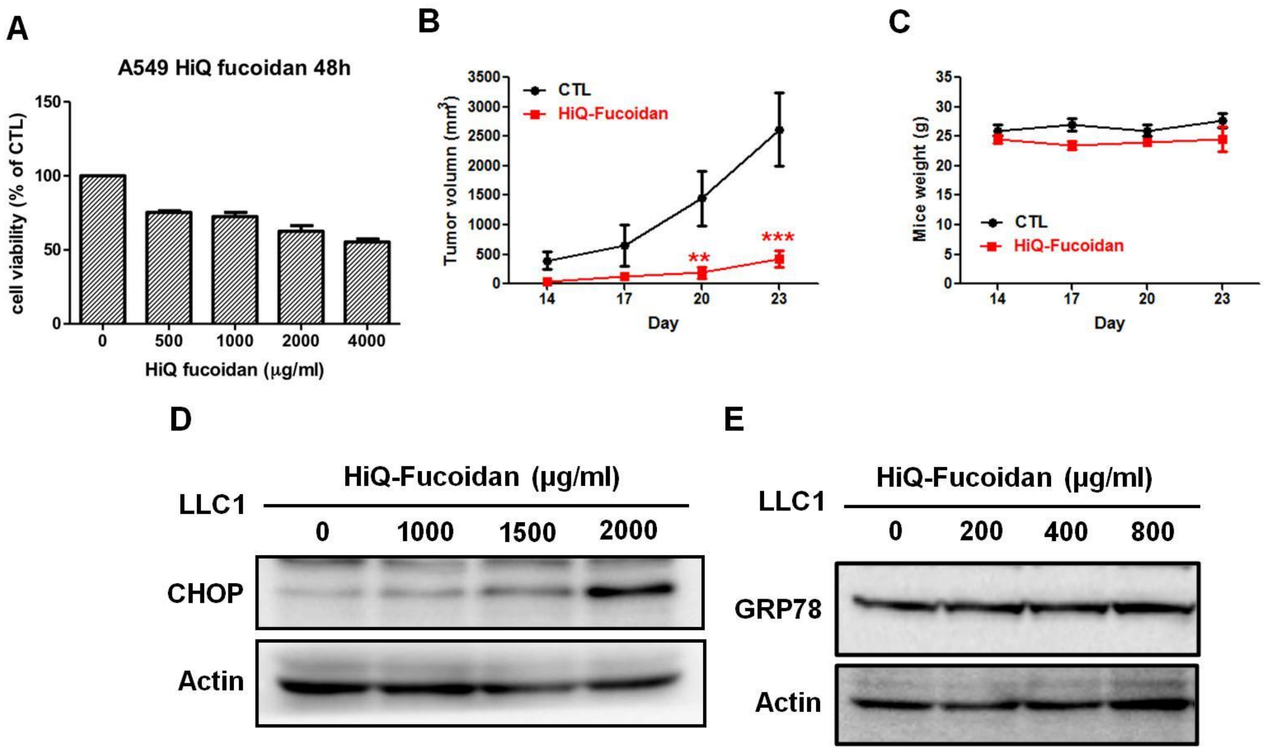

Supplementary Figure 7. HiQ-fucoidan suppresses tumor growth and induces apoptosis-related proteins *in vitro* and *in vivo*.

(A) A549 cells were treated with various doses of HiQ-fucoidan (μg/ml) for 48 h. Cell viabilities were determined by crystal violet staining assays. **(B)** Continuous once daily feeding of C57BL/6 male mice with HiQ-fucoidan (144 mg/kg) for 14 days prior to inoculation with LLC1 cells. After the mice were fed HiQ-fucoidan, LLC1 cells (2×10^5) were subcutaneously inoculated into the mouse abdomens, and the continuous once daily feeding of mice with HiQ-fucoidan was continued for 23 days. The tumor volumes were measured every 3 days for 23 days. **(C)** The body weights of the mice were measured every 3 days for 23 days. **(D-E)** LLC1 cells (5×10^5 cells) were treated with various dosages of HiQ-fucoidan (μg/ml) for 24 h. The treatment was followed by Western blotting analysis of whole-cell lysates to detect CHOP and GRP78 expression. Actin was used as an internal control.
